# Supplementary material for: Antimicrobial Susceptibility Testing Using the MYCO Test System and MIC Distribution of 8 Drugs against Clinical Isolates of Nontuberculous Mycobacteria from Shanghai
Source: Microbiol Spectr. 2023 Feb 21;11(2):e02549-22. doi: 10.1128/spectrum.02549-22 (PMC10100917; doi:10.1128/spectrum.02549-22)
Supplement: Supplemental file 1 — Supplemental material. Download spectrum.02549-22-s0001.pdf, PDF file, 0.05 MB [file spectrum.02549-22-s0001.pdf]

Table S1 The MICs of Bdq and Cfz against clinically isolated species of SGM with less than 10 isolates

			MIC(s) (µg/ml) by antimicrobial agent (no. of isolates)	
Category	species	No. of isolates	BDQ	CFZ
SGM	*M. intracellulare and M. avium complex*	1	<=0.12	0.12
	M. intracellulare and other mycobacteria complex	1	<=0.12	<=0.06
	M. kansasii and M. intracellulare complex	1	<=0.12	0.12
	M. kansasii and M. avium complex	1	<=0.12	<=0.06
	M. malmoense and M. avium complex	1	<=0.12	<=0.06
	M. malmoense and other mycobacteria	1	<=0.12	0.12
	M. gordonae	1	<=0.12	0.5
	M. flavescens	1	<=0.12	<=0.06
	M. szulgai	2	<=0.12 (2)	<=0.06 (2)
	other mycobacteria	4	<=0.12 (4)	<=0.06 (3), 0.12

(The number of isolates is in parentheses.)

Table S2 The MICs of Bdq and Cfz against clinically isolated species of RGM with less than 10 isolates

			MIC(s) (µg/ml) by antimicrobial agent (no. of isolates)	
Category	species	No. of isolates	BDQ	CFZ
RGM	*M. abscessus and M. intracellulare complex*	8	<=0.12 (7), 0.25	0.12 (4), 0.25 (4)
	M. abscessus and M. avium complex	2	<=0.12 (2)	0.12, 0.25
	M. intracellulare and other mycobacteria complex	1	>4	0.5
	M. fortuitum	2	<=0.12 (2)	0.12, 0.25
	other mycobacteria	1	<=0.12	0.25

(The number of isolates is in parentheses.)
